# The conserved poxvirus membrane entry-fusion apparatus component OPG147 targets MITA/STING for immune evasion

**DOI:** 10.1371/journal.ppat.1013198

**Published:** 2025-06-11

**Authors:** Xue-Mei Yi, Mi Li, Su-Yun Wang, Shi-Han Wang, Jia-Qing Zeng, Ya-Li Lei, Yu Zhang, Chun-Yu Zhu, Ying Zhang, Jun-Hui Song, Yun-Da Chen, Yun Wang, Hong-Bing Shu, Shu Li, Yan-Yi Wang

**Affiliations:** 1 Department of Infectious Diseases, Zhongnan Hospital of Wuhan University, Hubei Provincial Research Center for Basic Biological Sciences, Medical Research Institute, Frontier Science Center for Immunology and Metabolism, Taikang Center for Life and Medical Sciences, Wuhan University, Wuhan, Hubei, China; 2 State Key Laboratory of Virology and Biosafety, Wuhan Institute of Virology, Chinese Academy of Sciences, Wuhan, Hubei, China; 3 College of Life Science, Liaoning University, Shenyang, Liaoning, China; University of Arkansas for Medical Sciences, UNITED STATES OF AMERICA

## Abstract

Monkeypox virus (MPXV) causes severe diseases in immunocompromised individuals. How MPXV evades the host defense remain enigmatic. We performed expression screens and identified MPXV OPG147, a membrane fusion machinery protein, as an inhibitor of cGAS-MITA/STING-mediated innate immunity. OPG147 from other poxviruses including the prototypic vaccinia virus (VACV) shows similar functions. OPG147 is associated with MITA/STING and STIM1, a calcium sensor that retains MITA/STING in the ER. OPG147 does not block cGAMP binding to MITA, but inhibits its ISGylation, dimerization/oligomerization and trafficking, thereby suppressing its activation. Mutation of VACV OPG147 F55/T116/T117 to alanine (VACV^OPG147/3A^) has no effects on its infection and replication, but induces higher innate immune response compared with wild-type VACV in cells and mice. VACV^OPG147/3A^ infection also results in lower viral loads and decreased disease severity in mice. Our findings suggest that OPG147 contributes to immune evasion and is a virulence factor of poxviruses.

## Author summary

MPXV causes severe diseases in immunocompromised individuals. How MPXV evades the host defense remain enigmatic. We identified MPXV OPG147 as an inhibitor of cGAS-MITA/STING-mediated innate immunity. OPG147 from other poxviruses including VACV shows similar functions. OPG147 is associated with MITA/STING and STIM1, which retains MITA/STING in the ER. OPG147 suppresses MITA activation by inhibiting its ISGylation, dimerization/oligomerization, and trafficking. OPG147-mutanted VACV (VACV^OPG147/3A^) induces higher innate immunity in cells and mice. VACV^OPG147/3A^ infection also results in lower viral loads and decreased disease severity in mice. Our findings suggest that OPG147 contributes to immune evasion and is a virulence factor of poxviruses.

## Introduction

Monkeypox virus (MPXV) is a DNA virus that causes mpox, a zoonotic disease characterized by fever, headache, swollen lymph nodes, muscle aches, rash and even death in immunocompromised individuals [1]. The recent spread of mpox across multiple countries has prompted the World Health Organization (WHO) to declare it a Public Health Emergencies of International Concern (PHEICs) twice in the past three years [2]. Despite being the second most pathogenic poxviruses after Variola virus (VARV), how MPXV evades host defense and causes severe diseases are not well understood.

MPXV belongs to the *Orthopoxvirus genus* within the Poxviridae family, which also encompasses several other pathogens such as the prototypic vaccinia virus (VACV) and the lethal VARV. MPXV shares 96.3% nucleotide sequence identity with VACV [3], the prototypical poxvirus historically used as a smallpox vaccine and served as an excellent model for studying virus-host interactions [4]. Orthopoxviruses encode approximately 200 viral proteins, many of which are involved in virus-host interactions [5,6].

Poxviruses have evolved various strategies to evade the host immune defense, which contributes to its establishment of successful infection and pathogenesis. For examples, MPXV M2 protein subverts T cell activation and proliferation by interfering with the binding of CD28 and CTLA4 to their respective human B7.1/2 ligands [7]. The poxvirus poxin (also known as OPG188) has been identified as a family of 2′3′-cGAMP-specific nucleases, which cleaves 2′3′-cGAMP to antagonize the critical cGAS-MITA/STING innate immune pathway [8]. VACV 018 protein inhibits IFN-γ-induced signaling by blocking the association of STAT1 with IFN-γ receptor 1 (IFNGR1) [4]. VACV B8 and B18 proteins function as soluble IFN receptors to block antiviral activities triggered by type I and II IFNs respectively [9,10].

The cGAS-MITA/STING pathway is a central axis of innate immunity against DNA viruses, including poxviruses [11]. Upon binding of viral DNA, cGAS catalyzes the production of cGAMP with ATP and GTP as substrates [12–15]. cGAMP binds to the adaptor protein MITA (also named STING and ERIS) identified by us and others [16–19], which is physiologically retained at the endoplasmic reticulum (ER) by the calcium sensor stromal interaction molecule 1 (STIM1) in resting cells [20,21]. Binding of cGAMP to MITA causes its conformational changes, dimerization and interferon-stimulated gene 15 (ISG15) conjugation (ISGylation) [18,22–26]. ISGylation of MITA inhibits K48-linked polyubiquitination and degradation [27], while promotes its oligomerization and subsequent trafficking from the ER via Golgi apparatus to perinuclear punctuate structures [26–29]. It has been demonstrated that recruitment of the translocon-associated protein TRAPβ by iRhom2 to the MITA complex is critically involved in the process of trafficking of MITA [30]. During the trafficking of MITA, the kinase TBK1 and the transcription factor IRF3 are recruited to the MITA complex, at where TBK1 firstly phosphorylates MITA and IRF3, leading to induction of downstream antiviral genes [16,31,32].

To systematically reveal the mechanisms on how MPXV evades the cGAS-MITA pathway, we screened 196 MPXV-encoded proteins for their abilities to regulate cGAS-mediated signaling. These efforts identified MPXV OPG147, a critical component of the poxvirus membrane fusion apparatus essential for virus entry into cells and virus-induced cell-cell fusion [33,34]. We show that MPXV OPG147 is a conserved inhibitor of the cGAS-MITA innate immune signaling pathway. OPG147 inhibited ISG15 modification and oligomerization of MITA, while increased the association of MITA with STIM1, leading to inhibition of MITA trafficking and activation, and impairment of innate antiviral response. OPG147-mutanted VACV induced increased levels of IFN-β, exhibited diminished replication and caused attenuated pathogenesis in mice. Our findings unveil a conserved mechanism by which poxviruses antagonize MITA/STING to evade innate immunity.

## Results

### Identification of OPG147 as a conserved inhibitor of the cGAS-mediated pathway

**Fig 1:**
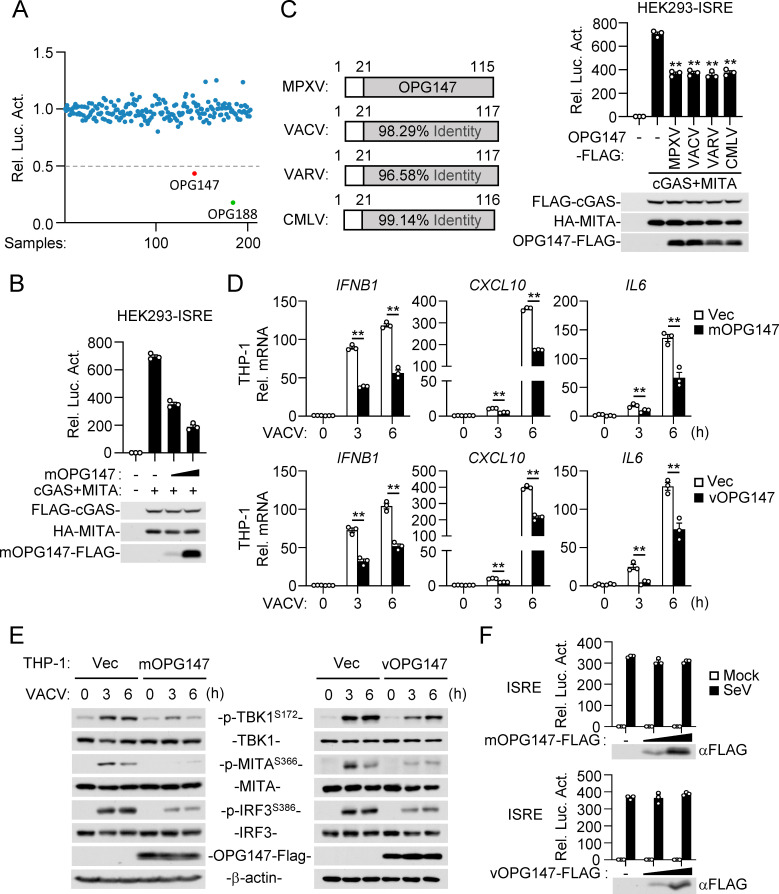
Identification of OPG147 as a conserved inhibitor of the cGAS-mediated pathway. (A) Screening of MPXV-encoded proteins that regulate the cGAS-mediated pathway. HEK293 cells (5 × 10^4^) were transfected with ISRE reporter plasmid (25 ng), expression plasmids for FLAG-cGAS (10 ng), HA-MITA (5 ng) and MPXV-encoded proteins (25 ng) or empty vector for 18 hours before luciferase assays. Data were normalized to empty vector-transfected control. (B) OPG147 inhibits cGAS-MITA-mediated ISRE activation in a dose-dependent manner. HEK293 cells (1 × 10^5^) were transfected with ISRE reporter plasmid (50 ng), expression plasmids for FLAG-cGAS (20 ng), HA-MITA (10 ng) and mOPG147-FLAG (0, 50, 100 ng) or empty vector for 18 hours before luciferase assays and immunoblotting analysis. Empty vector was added to ensure that each transfection receives the same amount of total DNA. (C) OPG147 is a conserved inhibitor of the cGAS-MITA pathway. Sequence identity of OPG147 among orthopoxviruses is shown (*Left*). HEK293 cells (1 × 10^5^) were transfected with ISRE reporter plasmid (50 ng), expression plasmids for FLAG-cGAS (20 ng), HA-MITA (10 ng), and FLAG-tagged OPG147 from different poxvirus species (50 ng) or empty vector for 18 hours before luciferase assays and immunoblotting analysis (*Right*). Empty vector was added to ensure that each transfection receives the same amount of total DNA. (D) Effects of OPG147 on transcription of downstream genes induced by VACV. The control THP-1 cells and THP-1 cells stably expressing mOPG147-FLAG or vOPG147-FLAG (1 × 10^6^) were left uninfected or infected with VACV (MOI = 1) for the indicated times before qPCR analysis. (E) Effects of OPG147 on phosphorylation of TBK1, MITA and IRF3 induced by VACV. The control THP-1 cells and THP-1 cells stably expressing mOPG147-FLAG or vOPG147-FLAG (1 × 10^6^) were left uninfected or infected with VACV (MOI = 1) for the indicated times before immunoblotting analysis with the indicated antibodies. (F) Effects of OPG147 on activation of ISRE induced by SeV. HEK293 cells (1 × 10^5^) were transfected with ISRE reporter plasmid (50 ng) and expression plasmids for mOPG147-FLAG or vOPG147-FLAG (0, 50, 100 ng) for 18 hours. The cells were then left untreated or treated with SeV (MOI = 1) for 12 hours before reporter assays and immunoblotting analysis with the indicated antibodies. Empty vector was added to ensure that each transfection receives the same amount of total DNA. Data shown in (B), (C), (D)&(F) are represented as mean ± SEM, n = 3 independent samples. These experiments were repeated for at least two times with similar results. * *P* < 0.05, ** *P* < 0.01.

To investigate the mechanisms by which MPXV evades the host innate immunity, we systematically screened 196 MPXV-encoded proteins for their abilities to regulate cGAS-MITA/STING-mediated signaling using ISRE (Interferon Stimulated Response Element) reporter assays. These screens identified MPXV-encoded protein OPG147 and OPG188 (also known as poxin in VACV) that markedly inhibited cGAS-MITA-mediated activation of ISRE in HEK293 cells (Fig 1A). It has been previously shown that viral poxins are cGAMP specific nucleases that inhibit cGAS-mediated signaling [8], therefore, we have focused our investigation on OPG147 in this study. Further experiments showed that OPG147 inhibited cGAS-MITA-mediated activation of ISRE in a dose dependent manner (Fig 1B) but had no marked effects on ISRE activation induced by TBK1 or IRF3 (S1A Fig). OPG147 is highly conserved across orthopoxviruses, such as MPXV, VACV, VARV and camelpox virus (CMLV) (Fig 1C). We compared the ability of OPG147 from different poxvirus species to regulate cGAS-MITA-mediated signaling and found that all the examined OPG147 proteins inhibited cGAS-MITA-mediated ISRE activation to a similar degree (Fig 1C). Subsequently, we generated human monocytic THP-1 cells overexpressing OPG147 derived from MPXV (mOPG147) or VACV (vOPG147) by lentivirus-mediated transduction. qPCR experiments indicated that ectopic expression of either mOPG147 or vOPG147 similarly inhibited VACV-induced transcription of *IFNB1*, *CXCL10*, and *IL6* genes in THP-1 cells (Fig 1D). In addition, OPG147 also inhibited transcription of *Ifnb1*, *Cxcl10*, and *Il6* genes induced by herring testis DNA (HT-DNA) in mouse lung fibroblasts (MLFs) (S1B Fig). Consistently, mOPG147 and vOPG147 also inhibited VACV-induced phosphorylation of TBK1, MITA and IRF3 in THP-1 cells, which is a hallmark of innate immune signaling following DNA virus infection (Fig 1E). These data suggest that OPG147 inhibits DNA virus-triggered downstream signaling events. In reporter assays, mOPG147 and vOPG147 had no marked effects on ISRE activation induced by infection of Sendai virus (SeV), which is a RNA virus (Fig 1F), suggesting that OPG147 specifically inhibits the cGAS-MITA pathway.

### OPG147-deficiency promotes innate immune response to MPXV and VACV

**Fig 2:**
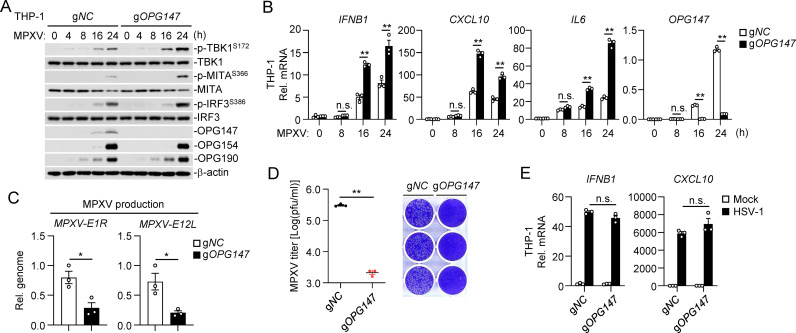
OPG147-deficiency promotes innate immune response to MPXV. (A) Effects of OPG147-deficiency on phosphorylation of TBK1, MITA and IRF3 induced by MPXV. The control and *OPG147* gRNA-edited THP-1 cells (1 × 10^6^) were left untreated or infected with MPXV (MOI = 0.1) for the indicated times before immunoblotting analysis with the indicated antibodies. (B) Effects of OPG147-deficiency on transcription of downstream genes induced by MPXV. The control and *OPG147* gRNA-edited THP-1 cells (1 × 10^6^) were left uninfected or infected with MPXV (MOI = 0.1) for the indicated times before qPCR analysis. (C&D) Effects of OPG147-deficiency on production of progeny viruses and cytopathic effects induced by these viruses. The control and *OPG147* gRNA-edited THP-1 cells (1 × 10^6^) were infected with MPXV (MOI = 0.1) for 2 hours. The cells were then washed twice with PBS, cultured in RPMI 1640 medium containing 2% FBS for 48 hours. Cells were then centrifuged, and the cell culture medium was collected. Titers of progeny viruses in the cell culture medium were quantified by qPCR (C) and plaque assays (D). (E) Effects of expression of the *OPG147* gRNA on transcription of downstream genes induced by HSV-1. The control and *OPG147* gRNA-edited THP-1 cells (1 × 10^6^) were left uninfected or infected with HSV-1 (MOI = 1) for the indicated times before qPCR analysis. Data shown in (B), (C), (D)&(E) are represented as mean ± SEM, n = 3 independent samples. All the experiments were repeated for at least two times with similar results. n.s., not significant. * P < 0.05, ** P < 0.01.

To further investigate the role of viral OPG147 in innate antiviral response, we generated a stable THP1 cell line expressing a gRNA that can target both *mOPG147* and *vOPG147* using the CRISPR-Cas9 system. Immunoblotting analysis indicated that knockout of mOPG147 markedly enhanced MPXV-induced phosphorylation of TBK1, MITA, and IRF3 in THP-1 cells (Fig 2A). In addition, knockout of mOPG147 also promoted MPXV-induced transcription of *IFNB1*, *CXCL10*, and *IL6* genes in THP-1 cells (Fig 2B). In these experiments, gRNA targeting of *OPG147* had no marked effects on expression of the viral early gene OPG190 and the entry gene OPG154 (Fig 2A), nor on transcription of the early genes OPG188 and OPG190 or the entry gene OPG154 (S2 Fig). We further examined the role of mOPG147 in MPXV replication by analyzing levels of MPXV *E1R* and *E12L* genes (equivalent to VACV *D1R* and *D12L*) at 48 hours post-infection. As shown in Fig 2C, production of MPXV progeny viruses was significantly reduced in the cell culture medium of *OPG147* gRNA-edited THP-1 cells. Correspondingly, the cytopathic effects caused by these progeny viruses were reduced compared to those from control gRNA-edited cells (Fig 2D). In similar experiments, knockout of vOPG147 promoted VACV-induced phosphorylation of TBK1, MITA and IRF3 (S3A Fig), transcription of *IFNB1*, *CXCL10*, and *IL6* genes (S3B Fig), and inhibited production of progeny viruses (S3C Fig) and cytopathic effects induced by the produced progeny viruses (S3D Fig). Expression of the *OPG147* gRNA had no significant effects on transcription of *IFNB1* and *CXCL10* genes induced by the DNA virus herpes simplex virus 1 (HSV-1) (Fig 2E). These results collectively suggest that OPG147-deficiency enhances innate immune response to MPXV and VACV in THP-1 cells.

### OPG147 is associated with MITA

**Fig 3:**
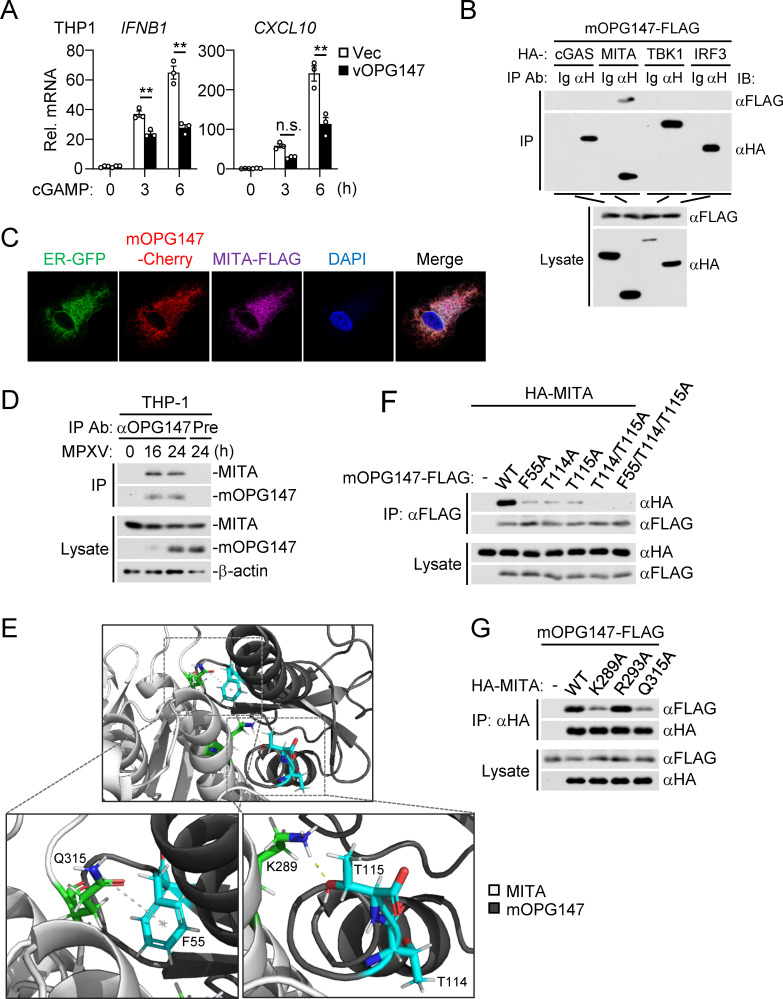
OPG147 is associated with MITA. (A) Effects of OPG147 on transcription of downstream genes induced by cGAMP. The control THP-1 cells and THP-1 cells stably expressing vOPG147 (1 × 10^6^) were left untreated or treated with 2′3′-cGAMP (100 ng/mL) for the indicated times before qPCR analysis. Data are represented as mean ± SEM, n = 3 independent samples. n.s., not significant. *P < 0.05; **P < 0.01. (B) mOPG147 is associated with MITA. HEK293 cells (5 × 10^6^) were transfected with the indicated plasmids. Eighteen hours post-transfection, co-immunoprecipitation was performed with control mouse IgG or anti-HA. The immunoprecipitates and lysates were analyzed by immunoblotting with the indicated antibodies. (C) mOPG147 is colocalized with MITA in the ER. Confocal microscopy of HT1080 cells transfected with ER-GFP, mOPG147-cherry and MITA-FLAG for 24 hours.(D) Viral OPG147 is associated with endogenous MITA following MPXV infection. THP-1 cells (3 × 10^7^) were infected with MPXV (MOI = 0.1) for the indicated times before co-immunoprecipitation and immunoblotting analysis. (E) Key interactions between MITA (light grey) and mOPG147 (dark grey). The inset highlights key residues involved in the interaction: Q315 of MITA corresponds to F55 of mOPG147, while K289 of MITA corresponds to T114 and T115 of mOPG147. (F-G) Association between mOPG147 or its mutants and MITA or its mutants. HEK293 cells (2 × 10^6^) were transfected with the indicated plasmids for 18 hours before co-immunoprecipitation and immunoblotting analysis with the indicated antibodies. All the experiments were repeated for at least two times with similar results.

Our earlier experiments showed that OPG147 inhibited VACV-induced phosphorylation of MITA and HT-DNA-induced transcription of downstream genes (Figs 1E and S1B), while knockout of OPG147 enhanced MPXV- and VACV-induced phosphorylation of MITA (Figs 2A and S3A). These results suggest that OPG147 functions at or upstream of MITA in the cGAS-MITA pathway. To further elucidate the molecular mechanisms underlying the inhibitory effects of OPG147 on innate antiviral response, we investigated its effects on cGAMP-induced signaling. The results indicated that ectopic expression of OPG147 inhibited cGAMP-induced transcription of *IFNB1* and *CXCL10* genes in THP1 cells (Fig 3A), suggesting that OPG147 acts downstream of cGAMP. Consistently, transient transfection and co-immunoprecipitation experiments indicated that both mOPG147 and vOPG147 were specifically associated with MITA but not with other examined components in the cGAS-MITA pathway, including cGAS, TBK1, and IRF3 (Figs 3B and S5A). Given that MITA is ER-localized, we investigated whether OPG147 colocalizes with MITA at the ER. Confocal microscopy indicated that both mOPG147 and vOPG147 colocalized with MITA at the ER (Figs 3C and S5B). To further confirm the association of OPG147 and endogenous MITA, we performed endogenous co-immunoprecipitation experiments following MPXV infection in THP-1 cells. The results indicated that mOPG147 was associated with endogenous MITA following MPXV infection (Figs 3D and S4). These results suggest that OPG147 is associated with MITA following MPXV infection.

### Identification of critical residues important for the interaction between OPG147 and MITA

To further understand the molecular basis of the OPG147-MITA interaction, we employed AlphaFold2 to predict the key interaction sites. This analysis identified the key interactions between MITA^Q315^ and mOPG147^F55^, and between MITA^K289^ and mOPG147^T114/T115^ (Fig 3E). Sequence analysis indicated that F55, T114, and T115 of mOPG147 are identical to F55, T116 and T117 of vOPG147 (S5C Fig). We then mutated these residues to alanine, either individually or in combination, and examined their effects on the interaction between OPG147 and MITA. Transient transfection and co-immunoprecipitation experiments indicated that mutation of F55, T114, or T115 of mOPG147, or F55, T116, or T117 of vOPG147, to alanine reduced their interaction with MITA. Simultaneous mutation of T114 and T115 of mOPG147, or T116 and T117 of vOPG147, to alanines further impaired this interaction, while simultaneous mutation of all three residues to alanines (3A) abolished the interaction (Figs 3F and S5D). Consistently, mutation of K289 or Q315 but not R293 of MITA to alanine markedly reduced its interaction with mOPG147 (Fig 3G) or vOPG147 (S5E Fig). These results suggest that F55, T114 and T115 of mOPG147, or F55, T116, and T117 of vOPG147, and Q315 and K289 of MITA are essential for the interaction between OPG147 and MITA.

### OPG147 inhibits ISG15 modification and oligomerization of MITA/STING

**Fig 4:**
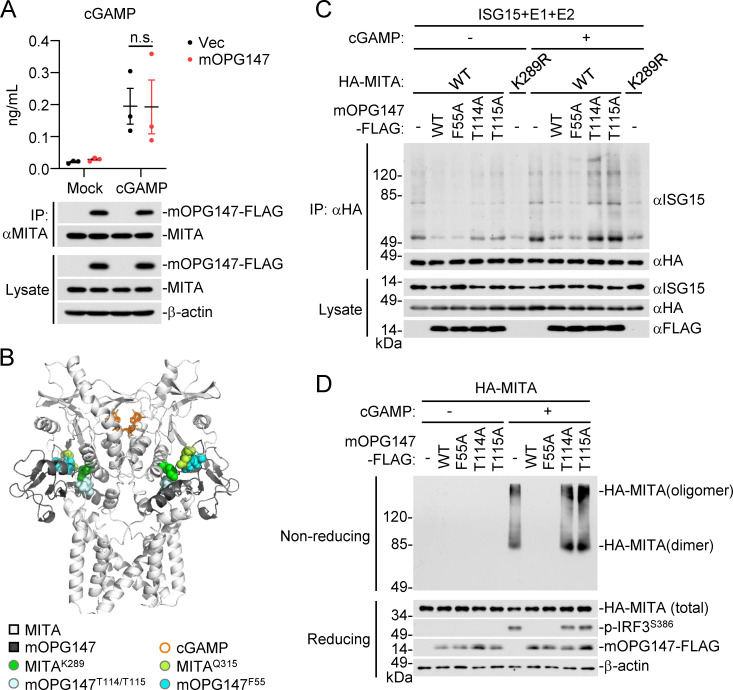
OPG147 inhibits ISGylation, dimerization and oligomerization of MITA. (A) OPG147 has no effects on binding of cGAMP to MITA. The control THP-1 cells and THP-1 cells stably expressing mOPG147-FLAG (3 × 10^7^) were lysed in lysis buffer, followed by co-immunoprecipitation with anti-MITA and Protein G Sepharose. The beads were incubated with 2′3′-cGAMP (1 μg/mL) in binding buffer, washed, and heated to remove denatured proteins. The heat-resistant supernatant was assayed for cGAMP levels by ELISA (histograph). A small aliquot of immunoprecipitates and lysates were analyzed by immunoblotting with the indicated antibodies (lower blots). Data are represented as ± SEM, n = 3 independent samples. n.s., not significant. (B) Binding of mOPG147 to MITA blocks ISGylation of MITA at K289. The image shows a structural model of a mOPG147-MITA complex, where mOPG147 (dark grey) interacts with the cGAMP (orange)-bound MITA homodimer (white). (C) Effects of OPG147 and its mutants on ISGylation of MITA. HEK293 cells (2 × 10^6^) were transfected with the indicated plasmids for 18 hours. The cells were then left untreated or treated with 2′3′-cGAMP (1 μM) for 30 min, and further incubated in medium for 1 hour before co-immunoprecipitation and immunoblotting analysis were performed with the indicated antibodies. (D) Effects of OPG147 and its mutants on dimerization and oligomerization of MITA. HEK293 cells (2 × 10^6^) were transfected with the indicated plasmids for 18 hours. The cells were then left untreated or treated with 2′3′-cGAMP (1 μM) for 30 min, and further incubated in medium for 1 hour before immunoblotting analysis were performed with the indicated antibodies. All the experiments were repeated for at least two times with similar results.

We next investigated how OPG147 inhibits MITA/STING-mediated innate immune signaling. We firstly determined whether OPG147 inhibits binding of MITA to cGAMP. We immunoprecipitated MITA from control or mOPG147-expressing THP1 cells and measured its ability to bind to cGAMP by ELISA. The results indicated that mOPG147 did not have marked effects on the amounts of cGAMP associated with MITA (Fig 4A). Consistently, structural docking analysis indicated that mOPG147 did not interfere with binding of MITA to cGAMP (Fig 4B). It has been reported that ISG15 modification (ISGylation) of MITA at K289 facilitates its oligomerization (26), which is crucial for MITA activation. Since K289 of MITA is crucial for the interaction of MITA with OPG147 (Figs 3G and S5E), we investigated the effects of OPG147 on ISGylation and oligomerization of MITA. We co-expressed ISG15, ubiquitin-activating enzyme E1-like protein (UBE1L, E1), ubiquitin-conjugating human enzyme 8 (UBCH8, E2), HA-MITA or HA-MITA^K289R^, and mOPG147 or its mutants with or without cGAMP stimulation, and performed co-immunoprecipitation and immunoblotting analysis to examine ISGylation of wild-type MITA and MITA^K289R^. cGAMP increased ISGylation of MITA, and cGAMP-induced ISGylation of MITA was markedly inhibited by wild-type mOPG147 and mOPG147^F55A^ but not mOPG147^T114A^ or mOPG147^T115A^ (Fig 4C), consistent with the results showing T114 and T115 of mOPG147 are important for its interaction with K289 of MITA (Fig 3F and 3G). Consistently, structural docking analysis confirmed that binding of mOPG147 to MITA completely blocks accessibility of potential ISG15 ligases to K289 of MITA (Fig 4B). Additionally, non-reducing gel electrophoresis and immunoblotting experiments indicated that wild-type mOPG147 and mOPG147^F55A^ but not mOPG147^T114A^ or mOPG147^T115A^ markedly decreased dimerization and oligomerization of MITA induced by cGAMP in HEK293 cells (Fig 4D). These findings suggest that OPG147 inhibits both ISGylation and oligomerization of MITA by blocking accessibility of ISG15 ligases to K289 of MITA.

### OPG147 impairs MITA trafficking by inhibiting its dissociation from STIM1

**Fig 5:**
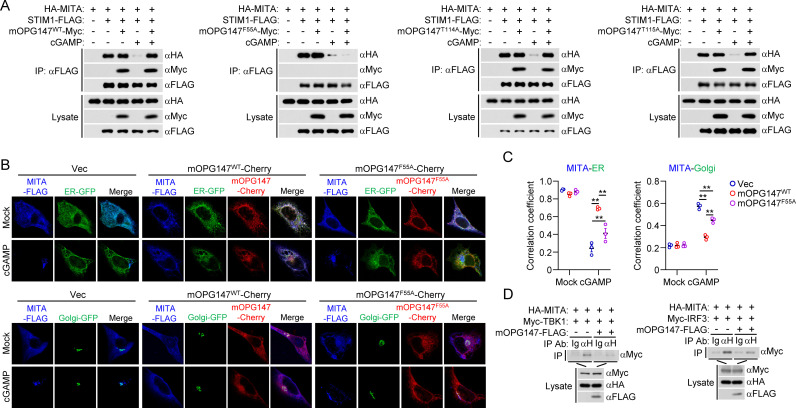
OPG147 blocks cGAMP-triggered dissociation of MITA from STIM1 and trafficking. (A) Effects of mOPG147 and its mutants on association of MITA with STIM1. HEK293 cells (2 × 10^6^) were transfected with the indicated plasmids for 18 hours. The cells were then left untreated or treated with 2′3′-cGAMP (1 μM) for 30 min, and further incubated in medium for 1 hour before co-immunoprecipitation and immunoblotting analysis were performed with the indicated antibodies. (B&C) mOPG147 impairs MITA trafficking. *Mita*^*-/-*^ MLFs reconstituted with MITA-FLAG, co-transfected with RAB9-GFP (for ER) or GM130-GFP (for Golgi) and mOPG147^WT^-Cherry or mOPG147^F55A^-Cherry, were stimulated with 2′3′-cGAMP (100 ng/mL) for 2 hours before confocal microscopy. Colocalization of RAB9-GFP or GM130-GFP with MITA under each condition was analyzed by calculation of Pearson’s correlation coefficients. Data show mean ± SEM of 3 cells per group (C) and one representative experiment (B). * P < 0.05, ** P < 0.01. (D) mOPG147 impairs recruitment of TBK1 and IRF3 to MITA. HEK293 cells (5 × 10^6^) were transfected with the indicated plasmids. Eighteen hours post-transfection, co-immunoprecipitation was performed with control mouse IgG or anti-HA. The immunoprecipitates and lysates were analyzed by immunoblotting with the indicated antibodies.

Previously, it has been shown that the ER-located calcium sensor STIM1 retains MITA at the ER in resting cells, and cGAMP binding to MITA triggers its dissociation from STIM1 and translocation from the ER via Golgi to perinuclear punctate structures, a process required for its recruitment and activation of TBK1 and IRF3 as well as induction of downstream antiviral genes [35,36]. We next investigated whether OPG147 affects this process. Co-immunoprecipitation experiments indicated that STIM1 was constitutively associated with MITA, and cGAMP stimulation caused dissociation of STIM1 and MITA (Fig 5A). STIM1 interacted with wild-type mOPG147, mOPG147^T114A^ and mOPG147^T115A^ but not mOPG147^F55A^, and overexpression of wild-type mOPG147, mOPG147^T114A^ and mOPG147^T115A^ but not mOPG147^F55A^ blocked cGAMP-induced dissociation of MITA from STIM1 (Fig 5A). Consistently, confocal microscopy showed that wild-type mOPG147 significantly impaired MITA trafficking from the ER via the Golgi apparatus to perinuclear punctate structures. Compared with wild-type mOPG147, mOPG147^F55A^ weakly reduced cGAMP-induced MITA trafficking (Fig 5B and 5C). Co-immunoprecipitation experiments indicated that mOPG147 inhibited recruitment of TBK1 and IRF3 to MITA (Fig 5D). We performed similar experiments with vOPG147 and its mutants, and obtained similar results (S6 Fig). These results suggest that OPG147 interacts with both MITA and STIM1, and inhibits cGAMP-triggered dissociation of MITA from STIM1 and its traffsicking.

### OPG147 contributes to poxvirus evasion of host defense

**Fig 6:**
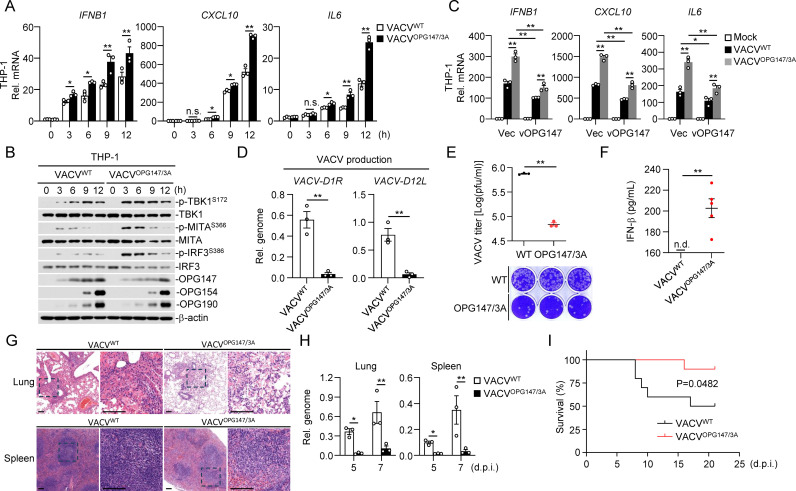
OPG147 contributes to VACV evasion of host defense and is a virulence factor *in vivo.* (A) Transcription of antiviral genes induced by wild-type VACV and VACV^OPG147/3A^. THP-1 cells (1 × 10^6^) were left uninfected or infected with wild-type VACV or VACV^OPG147/3A^ (MOI = 1) for the indicated times before qPCR analysis. (B) Phosphorylation of TBK1, MITA and IRF3 induced by wild-type VACV and VACV^OPG147/3A^. THP-1 cells (1 × 10^6^) were left uninfected or infected with wild-type VACV or VACV^OPG147/3A^ (MOI = 1) for the indicated times before immunoblotting analysis with the indicated antibodies. (C) The control vector- and vOPG147-FLAG-transduced THP1 cells (1 × 10^6^) were left un-infected or infected with wild-type VACV or VACV^OPG147/3A^ (MOI = 1) for the indicated times before qPCR analysis. (D&E) Replication and production of progeny viruses following infection of wild-type VACV and VACV^OPG147/3A^. THP-1 cells (1 × 10^6^) were infected with wild-type VACV or VACV^OPG147/3A^ (MOI = 0.1) for 2 hours. The cells were then washed twice with PBS, cultured in RPMI 1640 medium containing 2% FBS for 48 hours. The cells were centrifuged, and the cell culture medium was collected. Titers of progeny viruses in the cell culture medium were quantified by qPCR (D) and plaque assays (E). (F) ELISA analysis of IFN-β levels in BALF collected from C57BL/6J mice (n = 5 in each group) at 4 days post-infection with wild-type VACV or VACV^OPG147/3A^ at a dose of 2 × 10^6^ pfu per mouse. (G) Pathological analysis of mouse lungs and spleens. Lungs and spleens were harvested from C57BL/6J mice at 7 days post-infection with wild-type VACV or VACV^OPG147/3A^ at a dose of 2 × 10^6^ pfu per mouse for hematoxylin-eosin (HE) staining. Scale bars, 400 μm. (H) Measurement of viral loads in mouse lungs and spleens. VACV genomic copy numbers in lungs and spleens of mice infected with wild-type VACV or VACV^OPG147/3A^ (2 × 10^6^ pfu per mouse) were quantified by qPCR. (I) Survival of C57BL/6J mice (n = 10 in each group) infected with wild-type VACV or VACV^OPG147/3A^ (2 × 10^6^ pfu per mouse) was monitored daily for 21 days. Kaplan-Meier analysis was used to generate survival curves, and a log-rank test was used to compare groups. Data shown in (A), (C), (D), (E)&(H) are represented as mean ± SEM, n = 3 independent samples. All the experiments were repeated at least two times with similar results. n.s., not significant. n.d., not detectable. d.p.i., days post infiction. * P < 0.05, ** P < 0.01.

It has been reported that OPG147 (also known as VACWR140 or A21 in VACV) is a membrane fusion apparatus protein essential for virus entry into cells and virus-induced cell-cell fusion, and A21-deficient VACV exhibits defects in virus replication and spread (33). Given the utility of VACV as an excellent model for poxvirus-host interactions and the lack of suitable animal models for MPXV (Clade IIb) infection [4,37], we investigated the role of OPG147 in VACV evasion of host defense. We generated VACV mutants with a triple alanine substitution in vOPG147^F55/T116/T117A^ (VACV^OPG147/3A^) using the homologous recombination method (S7A Fig). Multiple cycle growth curves showed no significant differences in growth kinetics between wild-type VACV (VACV^WT^) and VACV^OPG147/3A^ in interferon-deficient Vero cells (S7B Fig), suggesting that these MITA-interacting residues of OPG147 are not important for its role in viral entry and virus-induced cell-cell fusion. qPCR experiments indicated that transcription of downstream antiviral genes such as *IFNB1*, *CXCL10*, and *IL6* induced by VACV^OPG147/3A^ was significantly increased compared to those induced by wild-type VACV in THP-1 cells (Fig 6A). Consistently, phosphorylation of TBK1, MITA and IRF3 was markedly increased following infection with VACV^OPG147/3A^ compared to wild-type VACV in THP-1 cells (Fig 6B). In these experiments, mutation of vOPG147 F55, T116 and T117 had no marked effects on expression of viral entry and early genes (Figs 6B and S7C). To further confirm the effects of OPG147 on innate immune response, we ectopically expressed vOPG147 and examined its effects on VACV^OPG147/3A^-induced transcription of downstream genes. The results indicated that ectopic expression of vOPG147 reversed the increased transcription of *IFNB1, CXCL10* and *IL6* genes induced by VACV^OPG147/3A^ in THP-1 cells (Fig 6C). We further compared replication of wild-type VACV and VACV^OPG147/3A^ by analyzing levels of its *D1R* and *D12L* genes at 48 hours post-infection. As shown in Fig 6D, production of progeny viruses was significantly reduced in the cell culture medium of THP-1 cells infected with VACV ^OPG147/3A^. Correspondingly, the viral titers and cytopathic effects caused by the progeny viruses were reduced compared to those from THP-1 cells infected with wild-type VACV (Fig 6E). These results suggest that OPG147 plays a critical role in antagonizing innate immune response to VACV.

### OPG147 is a virulence factor *in vivo*

To investigate the roles of OPG147 in VACV immune evasion and virulence *in vivo*, we performed intranasal infection experiments using wild-type VACV or VACV^OPG147/3A^ in C57BL-6J mice. We found that infection with VACV^OPG147/3A^ induced dramatically higher levels of IFN-β in bronchoalveolar lavage (BALF) in comparison to infection with wild-type VACV (Fig 6F). Hematoxylin-eosin (HE) staining analysis indicated that mice infected with wild-type VACV exhibited more serious pathological injury of the lung and spleen compared to those infected with VACV^OPG147/3A^ (Fig 6G). Infection of C57BL-6J mice with wild-type VACV led to significantly higher viral titers in the lung and spleen comparing to infection with VACV^OPG147/3A^ (Fig 6H). In addition, mice infected with wild-type VACV were more susceptible to death compared to those infected with VACV^OPG147/3A^ (Fig 6I). These results demonstrate that virulence of VACV^OPG147/3A^ is attenuated despite having similar replication capacity comparing to wild-type VACV, and suggest that OPG147 is a virulence factor.

## Discussion

Poxviruses are large DNA viruses that replicate exclusively in the cytoplasm of infected cells. They encode multiple immunomodulators to evade the host immune defense, which is crucial for their successful infection and pathogenesis [1]. The cGAS-MITA/STING pathway plays a critical role in host defense against poxvirus infection [11,38]. To systematically investigate whether the cGAS-MITA/STING pathway is targeted by MPXV proteins for immune evasion, we performed expression screens and identified MPXV OPG147 as a conserved inhibitor of the cGAS-MITA innate immune signaling pathway. OPG147 is a critical component of the poxvirus membrane fusion apparatus, which is essential for virus entry into cells and virus-induced cell-cell fusion, and its deficiency in VACV results in impaired virus replication and spread [33,34].

Our results reveal a conserved function of OPG147 on the cGAS-MITA innate immune signaling pathway. All examined OPG147 proteins from different poxvirus species inhibited cGAS-MITA-mediated ISRE activation to similar degrees, demonstrating the conserved function of OPG147. Overexpression of OPG147 inhibited cGAS-mediated activation of ISRE in a dose dependent manner but had no marked effects on SeV-induced ISRE activation, suggesting that OPG147 specifically inhibits the cGAS-MITA pathway. Ectopic expression of OPG147 inhibited VACV- and MPXV-induced phosphorylation of TBK1, MITA and IRF3, as well as transcription of downstream antiviral genes, whereas OPG147-deficiency had opposite effects.

Several lines of evidence suggest that OPG147 targets MITA to inhibit the antiviral innate immune response. Firstly, ectopic expression of OPG147 inhibited VACV-induced phosphorylation of MITA and transcription of downstream antiviral genes induced by HT-DNA and cGAMP, while knockout of OPG147 enhanced MPXV- and VACV-induced phosphorylation of MITA. Second, transient transfection and co-immunoprecipitation experiments indicated that OPG147 was associated with MITA but not with other examined components in the cGAS-MITA/STING pathway. Consistently, viral OPG147 was associated with endogenous MITA following MPXV infection. Third, confocal microscopy indicated that OPG147 colocalized with MITA at the ER. Additionally, structural docking analysis confirmed the OPG147-MITA interaction, and further identified that F55, T114 and T115 of mOPG147, or F55, T116 and T117 of vOPG147, and Q315 and K289 of MITA are essential for the interaction between OPG147 and MITA.

**Fig 7:**
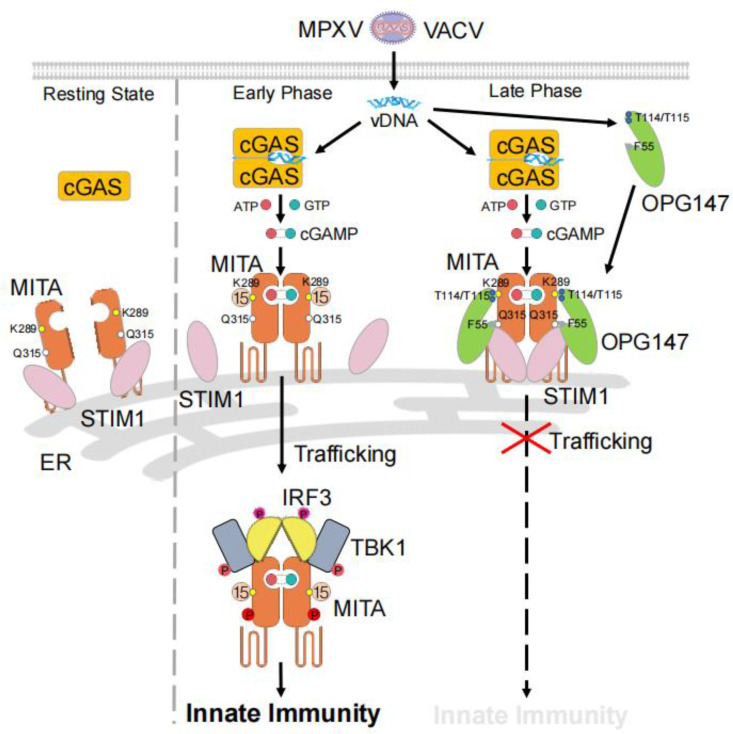
A model on OPG147-mediated evasion of the cGAS-MITA/STING pathway. See text for details.

Previously, it has been well established that MITA is retained at the ER by STIM1 in resting cells [20,21]. Upon binding of MITA to cGAMP, it undergoes ISGylation at K289, which prevents its K48-linked polyubiquitination and degradation, as well as promotes its oligomerization, dissociation from STIM1 and trafficking to perinuclear punctate structures [18,22–29]. In this process, TBK1 and IRF3 are recruited to MITA, leading to IRF3 activation and transcriptional induction of downstream antiviral genes. We performed a series of experiments to investigate the mechanisms on how OPG147 inhibits MITA activation. Our results indicated that OPG147 did not inhibit cGAMP binding to MITA. Instead, wild-type mOPG147 and mOPG147^F55A^ but not mOPG147^T114A^ or mOPG147^T115A^ markedly inhibited ISGylation of MITA, consistent with the results showing T114 and T115 of mOPG147 are important for its interaction with K289 of MITA as confirmed by structural docking analysis. Correspondingly, OPG147 also reduced dimerization and oligomerization of MITA induced by cGAMP. These results suggest that OPG147 inhibits ISGylation and dimerization/oligomerization of MITA by blocking accessibility of ISG15 ligases to K289 of MITA. In addition, wild-type mOPG147, mOPG147^T114A^ and mOPG147^T115A^ but not mOPG147^F55A^ interacted with STIM1, and inhibited dissociation of MITA from STIM1 following cGAMP stimulation. Consistently, OPG147 impaired cGAMP-induced trafficking of MITA from the ER via the Golgi apparatus to perinuclear punctate structures and assembly of the MITA-TBK1-IRF3 complex, which is important for the phosphorylation and activation of IRF3 and induction of downstream antiviral genes (Fig 7).

Given that MPXV shares 96.3% nucleotide sequence identity with VACV [3] and conserved mechanisms by which mOPG147 and vOPG147 evade the cGAS-MITA pathway, we generated a VACV mutant with a triple alanine substitution in vOPG147^F55/T116/T117A^ (VACV^OPG147/3A^) to investigate the role of OPG147 in immune evasion. Mutation of vOPG147 F55, T116 and T117 had no marked effects on expression of MPXV entry and early genes. Multiple cycle growth curves indicated that VACV^OPG147/3A^ had similar infection and replication capacity to wild-type VACV in IFN-deficient Vero cells. However, phosphorylation of cGAS downstream signaling components and transcription of downstream antiviral genes induced by VACV^OPG147/3A^ were markedly increased compared to those induced by wild-type VACV in THP-1 cells. Ectopic expression of wild-type vOPG147 reversed the increased transcription of *IFNB1, CXCL10* and *IL6* genes induced by VACV^OPG147/3A^ in THP-1 cells. The production of progeny viruses was significantly reduced in the cell culture medium of THP-1 cells infected with VACV^OPG147/3A^. Infection of C57BL-6J mice with VACV^OPG147/3A^ induced dramatically higher levels of IFN-β in BALF in comparison to infection with wild-type VACV. Mice infected with wild-type VACV were more susceptible to death and showed greater pathological injury and higher viral titers in the lung and spleen compared to those infected with VACV^OPG147/3A^, suggesting that OPG147 is a critical virulence factor.

Although certain VACV proteins, such as poxin and E5, have been reported to restrict the cGAS-MITA/STING pathway, OPG147 employs unique strategies to target MITA/STING activation. Poxin encoded by *B2R* gene cleaves 2′3′-cGAMP to restrict cGAS-MITA/STING signaling [8]. E5 targets cGAS to suppress DNA sensing [11]. These findings collectively suggest that poxviruses utilize diverse and distinct strategies to antagonize the cGAS-MITA/STING innate immune signaling pathway to ensure successful infection and pathogenesis.

In conclusion, we identified the conserved poxvirus membrane entry-fusion apparatus component OPG147 as a critical virulence factor and unveiled a conserved mechanism by which poxviruses antagonize MITA/STING to evade innate immunity. These findings contribute to our understanding of the mechanisms used by poxviruses to establish persistent infection and enhance our knowledge of the poxvirus-host interplay.

## Materials and methods

### Ethics statement

All animal experiments were approved by and performed in accordance with the guidelines of Wuhan University Animal Care and Use Committee (approval number: MRI2024-LACA39). All experiments with live MPXV were performed in the BSL-3 facilities of Wuhan Institute of Virology, Chinese Academy of Sciences (approval number: NBL3–3024037).

### Reagents, antibodies, cells, viruses and mice

RNAiso plus (Takara Bio, 9109), dual-specific luciferase assay kit (Promega, E1980), SYBR Green Mix (Bio-Rad, 172-5274), M-MLV reverse transcriptase (InvitroGen, 28025-013), RiboLock RNase inhibitor (Thermo Scientific, EO0381), lipofectamine 2000 (Invitrogen, 52887), 2’3’-cGAMP (InvivoGen, tlrl-nacga23-1), digitonin (Sigma-Aldrich, D141), puromycin (Amresco, J593), FBS (Cell Max, SA211.02), ELISA kits for murine IFN-β (PBL, 42400) and 2’3’-cGAMP (Cayman, 501700), and polybrene (Millipore, TR-1003-G) were purchased from the indicated companies.

Mouse antibodies against FLAG (Sigma, F3165), β-actin (Sigma, A2228), HA (OriGene, H6908); Rabbit antibodies against Myc (CST, 2278), TBK1 (Abcam, ab40647) and phospho-serine 172-TBK1 (Abcam, ab109272), MITA/STING (CST, 13647S), phospho-serine 366-MITA/STING (CST, 50907S) and phospho-serine 386-IRF3 (Abcam, ab76493), ISG15 (Proteintech, 15981-1-AP), STIM1 (Proteintech, 11565-1-AP), and MPXV B6R (also known as OPG190) (Antibody System, PVV13501) were purchased from the indicated manufacturers. Rabbit antisera against murine IRF3 were raised using the full-length recombinant protein as an antigen. Antisera against OPG147 were generated by immunizing mice with mOPG147 mRNA-LNP.

HEK293, THP-1 and HeLa cells were obtained from the American Type Culture Collection. BS-C-1 and HT1080 were obtained from China Center for Type Culture Collection. Reconstituted *Mita*^*-/-*^ mouse lymphatic fibroblast (MLF) cells were generated as previously described [30].

VACV (Western Reserve Strain, China Center for Type Culture Collection), MPXV (Clade IIb, CSTR:16533.06. IVCAS 6.9141, National Biosafety Laboratory, Wuhan, Chinese Academy of Sciences), HSV-1 (KOS strain, China Center for Type Culture Collection) and SeV (Cantell strain, Charles River Laboratories) were obtained from the indicated resources.

### Constructs

The expression clones encoding 196 independent MPXV-encoded proteins were constructed by standard molecular biology technique. Expression plasmids for FLAG- or Myc-tagged mOPG147 and its mutants; FLAG- or Myc-tagged vOPG147 and its mutants; mOPG147-cherry, mOPG147^F55A^-cherry, vOPG147-cherry, STIM1-FLAG, Myc- tagged TBK1 and IRF3 were constructed by standard molecular biology techniques. Expression plasmids for ISG15, UBE1L (E1) and UBCH8 (E2) were purchased from Origene. HA-tagged cGAS, MITA and its mutants, TBK1 and IRF3, FLAG-tagged cGAS and MITA, GFP-tagged RAB9 (ER marker), GM130 (Golgi marker) and the IRF1 and ISRE luciferase reporter plasmids were previously described [30,39–41].

### Stable cell lines

HEK293 cells were transfected with the indicated retroviral plasmids, along with pGag-pol and pVSV-G packaging plasmids by calcium phosphate precipitation. Twelve hours post-transfection, the culture medium was replaced with fresh antibiotic-free medium. After an additional 48 hours, the harvested viruses were used to infect THP-1 cells in the presence of polybrene (8 µg/mL). The infected THP-1 cells were selected with puromycin (2 µg/mL) for 6 days before experiments.

### CRISPR-Cas9 knockout

The protocols for genome engineering using the CRISPR/Cas9 system were previously described [42,43]. Briefly, double-stranded oligonucleotides corresponding to the target sequences were cloned into the lenti CRISPR-V2 vector. This vector was then co-transfected with packaging plasmids (pCMV-VSV-G and psPAX2) into HEK293 cells. Twelve hours post-transfection, the culture medium was replaced with fresh antibiotic-free medium. After an additional 48 hours, the viruses were harvested and used to infect THP-1 cells, which were subsequently selected with puromycin (2 μg/ml) for 6 days. The following gRNA sequences were used:

g*OPG147* #1: 5’- ACGTTTGTAGTTTACGTATG-3’;

g*OPG147* #2: 5’- ACCTCGTTTATCTTGGAACA-3’.

### Transfection and reporter assays

HEK293 cells were transfected using calcium phosphate precipitation. HT1080 and MLF cells were transfected with Lipofectamine 2000 according to the manufacturer’s instructions. pRL-TK (*Renilla* luciferase) reporter plasmid (0.01 μg) was included in each transfection to normalize for transfection efficiency. Empty control plasmid was added to ensure equal amounts of total DNA in each transfection. Luciferase assays were performed using a Dual-Specific Luciferase Assay Kit, and Firefly luciferase activities were normalized against *Renilla* luciferase activities.

### Co-immunoprecipitation and immunoblot analysis

For transient transfection and co-immunoprecipitation experiments, transfected HEK293 cells were lysed in Triton X-100 lysis buffer (20 mM Tris-HCl [pH 7.4], 150 mM NaCl, 1 mM EDTA, 1% Triton X-100, 10 μg/ml aprotinin, 10 μg/ml leupeptin, and 1 mM phenylmethylsulfonylfluoride). Co-immunoprecipitation and immunoblotting analysis were performed as previously described [44]. For endogenous co-immunoprecipitation experiments, the indicated cells were infected with MPXV for the indicated times or left uninfected before co-immunoprecipitation and immunoblotting analysis.

### RT-qPCR

The protocols for RT-qPCR were previously described [45]. Briefly, total RNA was extracted from cells using RNAiso plus reagent, followed by reverse transcription to generate cDNA. qPCR analysis was then performed to measure mRNA levels of the tested genes. Data shown are the relative abundance of the indicated mRNA normalized to that of human *ACTB*, except for viral titer data obtained from cell culture medium. qPCR was performed using the following primers:

Human *ACTB*: 5′-CACCATTGGCAATGAGCGGTTC-3′ (forward) and 5′-AGGTCTTTGCGGATGTCCACGT-3′ (reverse);

Human *IFNB1*: 5′-TTGTTGAGAACCTCCTGGCT-3′ (forward), 5′-TGACTATGGTCCAGGCACAG-3′ (reverse);

Human *CXCL10*: 5′-GGTGAGAAGAGATGTCTGAATCC-3′ (forward), 5′-GTCCATCCTTGGAAGCACTGCA-3′ (reverse);

Human *IL6*: 5′-AGACAGCCACTCACCTCTTCAG-3′ (forward) and 5′-TTCTGCCAGTGCCTCTTTGCTG-3′ (reverse);

*vOPG147*: 5′-TGACCTCGTTTATCTTGGAACATGGAAT-3′ (forward) and 5′- GAGACGCGAACGAGCCGCATA-3′ (reverse);

*mOPG147*: 5′-TGACCTCGTTTATCTTGGAACATGGAAT-3′ (forward) and 5′- GAGACGAGAACGAGCCGCATA-3′ (reverse);

MPXV *E1R*: 5′-TAGGATCTGGCGCTCAATCT-3′ (forward) and 5′- GGTGCGTTAATAGGTGGAGA-3′ (reverse);

MPXV *E12L*: 5′-GCCGACCGACATGTTAAAACT-3′ (forward) and 5′- GTTTATTGCGTGCGCTGAGGT-3′ (reverse);

MPXV *OPG188*: 5′-GTTTTACGCACACGCTTTCG-3′ (forward) and 5′- CTGACATCATTGGCAACCGTC-3′ (reverse);

MPXV *OPG190*: 5′-TGATGGTCCCGACGATGAGA-3′ (forward) and 5′- CACCCATAATTGTCAACGCCA-3′ (reverse);

MPXV *OPG154*: 5′-GCTTCGCGTTTAGTCTCTGG -3′ (forward) and 5′- TGGACGGAACTCTTTTCCCC-3′ (reverse);

VACV *D1R*: 5′-TAGGATCCGGTGCCCAGTCT-3′ (forward) and 5′- GGCGCGTTAATAGGCGGAGA-3′ (reverse);

VACV *D12L*: 5′-GCCGACCGACATGTTAAAACT-3′ (forward) and 5′- GTTTATTGCGTGCGCTGAGGT-3′ (reverse);

VACV *OPG188*: 5′-CGATGTTTTACGCACACGCT-3′ (forward) and 5′- CCCGATTCCGCTTAGAGCAT-3′ (reverse);

VACV *OPG190*: 5′-GGATCCAAATGCTGTCTGCG-3′ (forward) and 5′- CGCCGTTGCAACTTAGTGTC-3′ (reverse);

VACV *OPG154*: 5′-TGGACGGAACTCTTTTCCCC-3′ (forward) and 5′- GCCTCTGGCTTTTTAGCAGC-3′ (reverse).

### Confocal microscopy

HT1080 cells and *Mita*^*-/-*^ MLFs reconstituted with MITA-FLAG were transfected with the indicated plasmids using Lipofectamine 2000. Eighteen hours post-transfection, *Mita*^*-/-*^ MLFs reconstituted with MITA-FLAG were left untreated or treated with 2′3′-cGAMP (100 ng/mL) for 2 hours. Cells were fixed with 4% paraformaldehyde for 30 minutes and then washed three times with phosphate-buffered saline (PBS). Subsequently, cells were permeabilized and blocked in PBS containing 100 μM digitonin, 1% bovine serum albumin, 0.1% Tween 20 and 22.52 mg/mL glycine for 60 minutes, followed by three washes with PBS. Cells were then stained with the indicated primary and secondary antibodies. Nuclei were stained with DAPI for 2 minutes and washed three times with PBS. Finally, the cells were imaged using Leica Stellaris 5 WLL confocal microscope under a 63X oil objective.

### ISGylation analysis

To analyze ISGylation of MITA, HEK293 cells were transfected with the indicated plasmids for 18 hours. The cells were then left untreated or treated with 2’3’-cGAMP (1 μM) for 30 minutes, followed by a 1-hour incubation in fresh medium. The cells were harvested and lysed in 1 mL lysis buffer on ice for 30 minutes. The lysates were centrifuged at 12,000 rpm for 10 minutes at 4°C. The supernatant was aliquoted for co-immunoprecipitation with the indicated antibody and Protein G Sepharose (35 μL of a 1:1 slurry) at 4°C for 2 hours. The beads were washed three times with 1 mL lysis buffer containing 0.5 M NaCl. The bead-associated proteins were separated by SDS-PAGE and analyzed by immunoblotting with the indicated antibodies.

### Analyses of MITA dimerization and oligomerization

HEK293 cells were transfected with the indicated plasmids for 18 hours. The cells were then left untreated or treated with 2’3’-cGAMP (1 μM) for 30 minutes, followed by a 1-hour incubation in fresh medium. The cells were harvested and lysed in mild lysis buffer (20 mM Tris-HCl [pH 7.4], 150 mM NaCl, 1 mM EDTA, 1% Triton X-100, 10% glycerol, and a mixture of protease and phosphatase inhibitors) on ice for 15 minutes. Each sample was mixed with 6 × SDS loading buffer without 2-mercaptoethanol (2-ME) and sonicated to fragment the DNA. The samples were then clarified by centrifugation. The supernatant, without denaturing reagents, was separated by 10% SDS-PAGE and then subjected to immunoblotting to detect MITA dimerization and oligomerization. The remaining supernatant was treated with 2-ME, heated at 95°C for 15 minutes, and then separated by 10% SDS-PAGE to detect the expression of transfected plasmids.

### 2’3’-cGAMP binding assay

The control THP-1 cells and THP-1 cells stably expressing mOPG147-FLAG were lysed in lysis buffer (20 mM Tris-HCl [pH 7.4], 150 mM NaCl, 1 mM EDTA, 1% Triton X-100, 10 μg/ml leupeptin, 10 μg/ml aprotinin, 1 mM phenylmethylsulfonyl fluoride). The lysates were centrifuged at 12,000 rpm at 4°C for 10 minutes, and the supernatant was aliquoted for co-immunoprecipitation with anti-MITA and Protein G Sepharose (50 μL of a 1:1 slurry) at 4°C for 4 hours. A small aliquot of beads was removed for immunoblotting after centrifugation at 3000 rpm at 4°C for 2 minutes and three washes with lysis buffer containing 0.5 M NaCl. The remaining beads were incubated with 2′3′-cGAMP (1 μg/mL) in binding buffer (20 mM Tris-HCl [pH 7.4], 200 mM NaCl) for 15 minutes at room temperature. The beads were washed three times with 1mL lysis buffer containing 0.5 M NaCl, followed by three additional washes with 1 mL PBS. The bead-associated proteins in PBS were heated at 95°C for 10 minutes to remove denatured proteins. The heat-resistant supernatant was assayed for cGAMP levels by ELISA.

### VACV infection of mice

C57BL/6J female mice (6 weeks old) were anesthetized and intranasally infected with wild-type VACV or VACV^OPG147/3A^ at a dose of 2 × 10^6^ pfu per mouse. Mice were monitored daily for survival over a 21-day period. Bronchoalveolar lavage fluid (BALF) was collected from C57BL/6J mice at 4 days post-infection to measure IFN-β levels by ELISA. Lungs and spleens were harvested 7 days post-infection for pathological analysis by hematoxylin and eosin (HE) staining and measurement of viral titers by qPCR.

### Histology

Lungs and spleens were harvested from the indicated mice, washed with PBS, and immediately fixed in 4% paraformaldehyde. Paraffin-embedded sections of the indicated tissues were subjected to HE staining and then examined by light microscopy (Leica).

### ELISA

IFN-βlevels in BALF of infected mice were measured using a mouse IFN beta ELISA kit (Pestka Biomedical Laboratories).

### Generation of recombinant virus

To generate recombinant VACV^OPG147/3A^ virus, HeLa cells were transfected with pUC18-vOPG147^3A^-FLAG plasmids containing homologous arms using Lipofectamine 2000.

Four hours post-transfection, the culture medium was replaced with fresh FBS-free medium. The cells were then infected with wild-type VACV (MOI = 1) for 2 hours, followed by changing to fresh medium containing 2% FBS for continued culture. Homologous recombination between plasmid DNA and VACV genome resulted in replacement of *OPG147* gene with *OPG147*^*3A*^*-FLAG* in the viral genome. The cells were harvested 3 days post-infection and subjected to three cycles of freeze-thaw to release the virus. HeLa cells were infected with serial dilutions of the virus in 96-well plates and cultured for 10 days before PCR analyses and DNA sequencing. To obtain a pure recombinant VACV^OPG147(3A)^ strain, 4–5 rounds of screening were performed as described previously [46].

### Docking

Structural docking of mOPG147 onto MITA was performed using AlphaFold 2 [47,48]. A single mOPG147 molecule was docked onto MITA using default parameters. The C-terminal residues T114 and T115 of mOPG147 were positioned near K289 of MITA, and F55 of mOPG147 was positioned near Q315 of MITA. The close proximity of H in K289 to O in T115 (~1.9 Å) suggests a potential hydrogen bond interaction between these residues. mOPG147 was then symmetrically placed on both subunits of the MITA dimer (PDB ID: 8IK3) [49].

### Statistics

Statistical analysis was performed using Student’s unpaired t-test or two-way ANOVA with Prism GRAGHPAD 10. For the mouse survival study, Kaplan-Meier survival curves were generated and analyzed by Log-Rank test.

## Supporting information

S1 Fig OPG147 has no effect on TBK1 or IRF3-mediated ISRE activation and HT-DNA-induced signaling. (A) Effects of mOPG147 on TBK1 or IRF3-mediated ISRE activation. HEK293 cells (1 × 10^5^) were transfected with ISRE reporter plasmid (50 ng), expression plasmids for HA-TBK1 (100 ng) or HA-IRF3 (50 ng) and mOPG147-FLAG (0, 50, 100 ng) or empty vector for 18 hours before luciferase assays and immunoblotting analysis. Empty vector was added to ensure that each transfection receives the same amount of total DNA. (B) Effects of mOPG147 on transcription of downstream genes induced by HT-DNA. The control MLF cells and MLF cells stably expressing mOPG147-FLAG (1 × 10^6^) were left untreated or treated with HT-DNA (2 mg/mL) for 2 hours before qPCR analysis. Data shown in (A)&(B) are represented as mean ± SEM, n = 3 independent samples. All the experiments were repeated for at least two times with similar results. * P < 0.05, ** P < 0.01.
(TIFF)

S2 Fig The presence of the gRNA targeting *OPG147* has no effect on transcription of viral entry and early genes. The control and *OPG147* gRNA-edited THP-1 cells (1 × 10^6^) were left uninfected or infected with MPXV (MOI = 0.1) for the indicated times before qPCR analysis.Data are represented as mean ± SEM, n = 3 independent samples. All the experiments were repeated for at least two times with similar results. n.s., not significant.
(TIFF)

S3 Fig vOPG147-deficiency promotes innate immune response to VACV. (A) Effects of OPG147-deficiency on phosphorylation of TBK1, MITA and IRF3 induced by VACV. The control and *OPG147* gRNA-edited THP-1 cells (1 × 10^6^) were left uninfected or infected with VACV (MOI = 1) for the indicated times before immunoblotting analysis with the indicated antibodies. (B) Effects of OPG147-deficiency on transcription of downstream genes induced by VACV. The control and *OPG147* gRNA-edited THP-1 cells (1 × 10^6^) were left uninfected or infected with VACV (MOI = 1) for the indicated times before qPCR analysis. (C&D) Effects of OPG147-deficiency on production of progeny viruses. The control and *OPG147* gRNA-edited THP-1 cells (1 × 10^6^) were infected with VACV (MOI = 0.1) for 2 hours. The cells were then washed twice with PBS, cultured in RPMI 1640 medium containing 2% FBS for 48 hours. The cells were then centrifuged, and the cell culture medium was collected. Titers of progeny viruses in the cell culture medium were quantified by qPCR (C) and plaque assays (D). Data shown in (B), (C) & (D) are represented as mean ± SEM, n = 3 independent samples. All the experiments were repeated for at least two times with similar results. n.s., not significant. * P < 0.05, ** P < 0.01.
(TIFF)

S4 Fig Validation of OPG147 antibody. HEK293 cells (5 × 10^6^) were transfected with expression plasmids for mOPG147-FLAG or empty vector. Eighteen hours post-transfection, co-immunoprecipitation was performed with anti-FLAG. The immunoprecipitates and lysates were analyzed by immunoblotting with the indicated antibodies.
(TIFF)

S5 Fig vOPG147 is associated with MITA. (A) vOPG147 is associated with MITA. HEK293 cells (5 × 10^6^) were transfected with the indicated plasmids. Eighteen hours post-transfection, co-immunoprecipitation was performed with control mouse IgG or anti-HA. The immunoprecipitates and lysates were analyzed by immunoblotting with the indicated antibodies. (B) vOPG147 is colocalized with MITA in the ER. Confocal microscopy of HT1080 cells transfected with ER-GFP, vOPG147-Cherry and MITA-FLAG for 24 hours. (C) Alignment of OPG147 from MPXV and VACV. Key residues involved in its interaction with MITA are highlighted in red and marked with asterisks.
(TIFF)

S6 Fig vOPG147 impairs cGAMP-induced dissociation of MITA from STIM1 and its trafficking. (A) Effects of vOPG147 and its mutants on association of MITA with STIM1. HEK293 cells (2 × 10^6^) were transfected with the indicated plasmids for 18 hours. The cells were untreated or treated with 2′3′-cGAMP (1 mM) for 30 min and further incubated in medium for 1 hour before co-immunoprecipitation and immunoblotting analysis were performed with the indicated antibodies. (B) vOPG147 impairs MITA trafficking. *Mita*^*-/-*^ MLFs reconstituted with MITA-FLAG, ER-GFP and vOPG147-Cherry were stimulated with 2′3′-cGAMP (100 ng/mL) for 2 hours before confocal microscopy. (C) vOPG147 impairs recruitment of TBK1 and IRF3 to MITA. HEK293 cells (5 × 10^6^) were transfected with the indicated plasmids for 18 hours before co-immunoprecipitation and immunoblots were performed with the indicated antibodies. (D) Association between MITA and vOPG147 or its mutants. HEK293 cells (2 × 10^6^) were transfected with the indicated plasmids for 18 hours before co-immunoprecipitation and immunoblotting analysis with the indicated antibodies. (E) Association between vOPG147 and MITA or its mutants. HEK293 cells (2 × 10^6^) were transfected with the indicated plasmids for 18 hours before co-immunoprecipitation and immunoblotting analysis with the indicated antibodies. All the experiments were repeated for at least two times with similar results.
(TIFF)

S7 Fig Mutation of vOPG147 F55, T116 and T117 does not affect replication and production of VACV. (A) Sequencing traces confirmed alanine substitutions at residues F55, T116, and T117 of OPG147 in VACV. The recombinant VACV was verified by PCR amplification of its genomic DNA followed by DNA sequencing. (B) Multiple cycle growth curves of wild-type VACV and VACV^OPG^ 147^/^ 3^A^ in Vero cells. Vero cells (1 × 10^6^) were infected with wild-type VACV or VACV^OPG^ 147^/^ 3^A^ (MOI = 0.05) for 2 hours. The cells were washed twice with PBS and cultured in MEM medium containing 2% FBS. The cells and cell culture medium were harvested at the indicated time points, subjected to three freeze-thaw cycles, and then quantified for viral titers by plaque assays using BS-C-1 cells. (C) Effects of mutation of vOPG147 F55, T116 and T117 on transcription of entry and early genes of VACV. THP-1 cells (1 × 10^6^) were left uninfected or infected with wild-type VACV or VACV^OPG^ 147^/^ 3^A^ (MOI = 1) for the indicated times before qPCR analysis. Data are represented as mean± SEM, n = 3 independent samples. n.s., not significant.
(TIFF)
