# Supplementary figures and images for: The conserved poxvirus membrane entry-fusion apparatus component OPG147 targets MITA/STING for immune evasion
Source: PLoS Pathog. 2025 Jun 11;21(6):e1013198. doi: 10.1371/journal.ppat.1013198 (PMC12157864; doi:10.1371/journal.ppat.1013198)

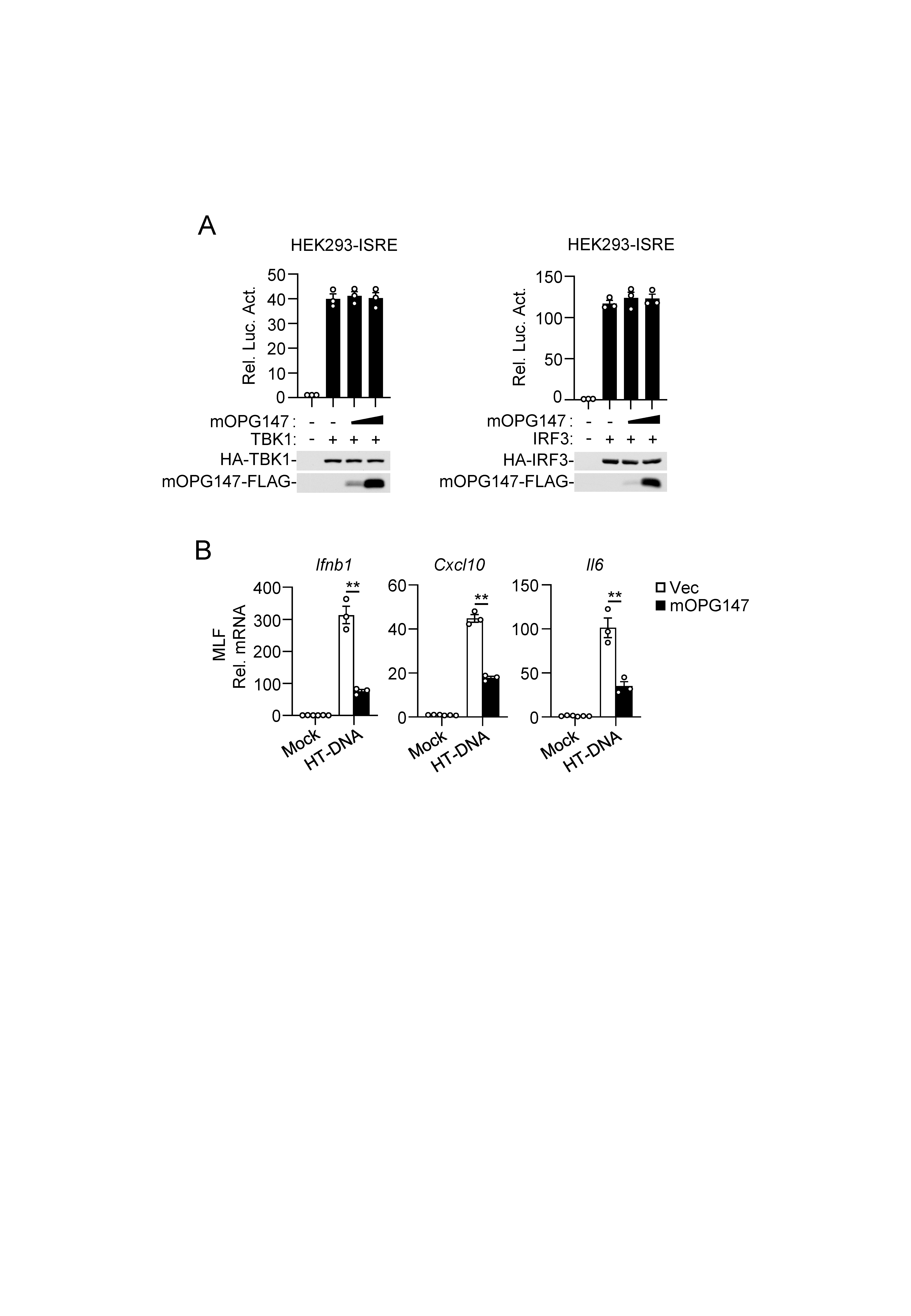

Supplement: S1 Fig — (A) Effects of mOPG147 on TBK1 or IRF3-mediated ISRE activation. HEK293 cells (1 × 10^5^) were transfected with ISRE reporter plasmid (50 ng), expression plasmids for HA-TBK1 (100 ng) or HA-IRF3 (50 ng) and mOPG147-FLAG (0, 50, 100 ng) or empty vector for 18 hours before luciferase assays and immunoblotting analysis. Empty vector was added to ensure that each transfection receives the same amount of total DNA. (B) Effects of mOPG147 on transcription of downstream genes induced by HT-DNA. The control MLF cells and MLF cells stably expressing mOPG147-FLAG (1 × 10^6^) were left untreated or treated with HT-DNA (2 mg/mL) for 2 hours before qPCR analysis. Data shown in (A)&(B) are represented as mean ± SEM, n = 3 independent samples. All the experiments were repeated for at least two times with similar results. * P < 0.05, ** P < 0.01. (TIFF) [file ppat.1013198.s001.tiff]

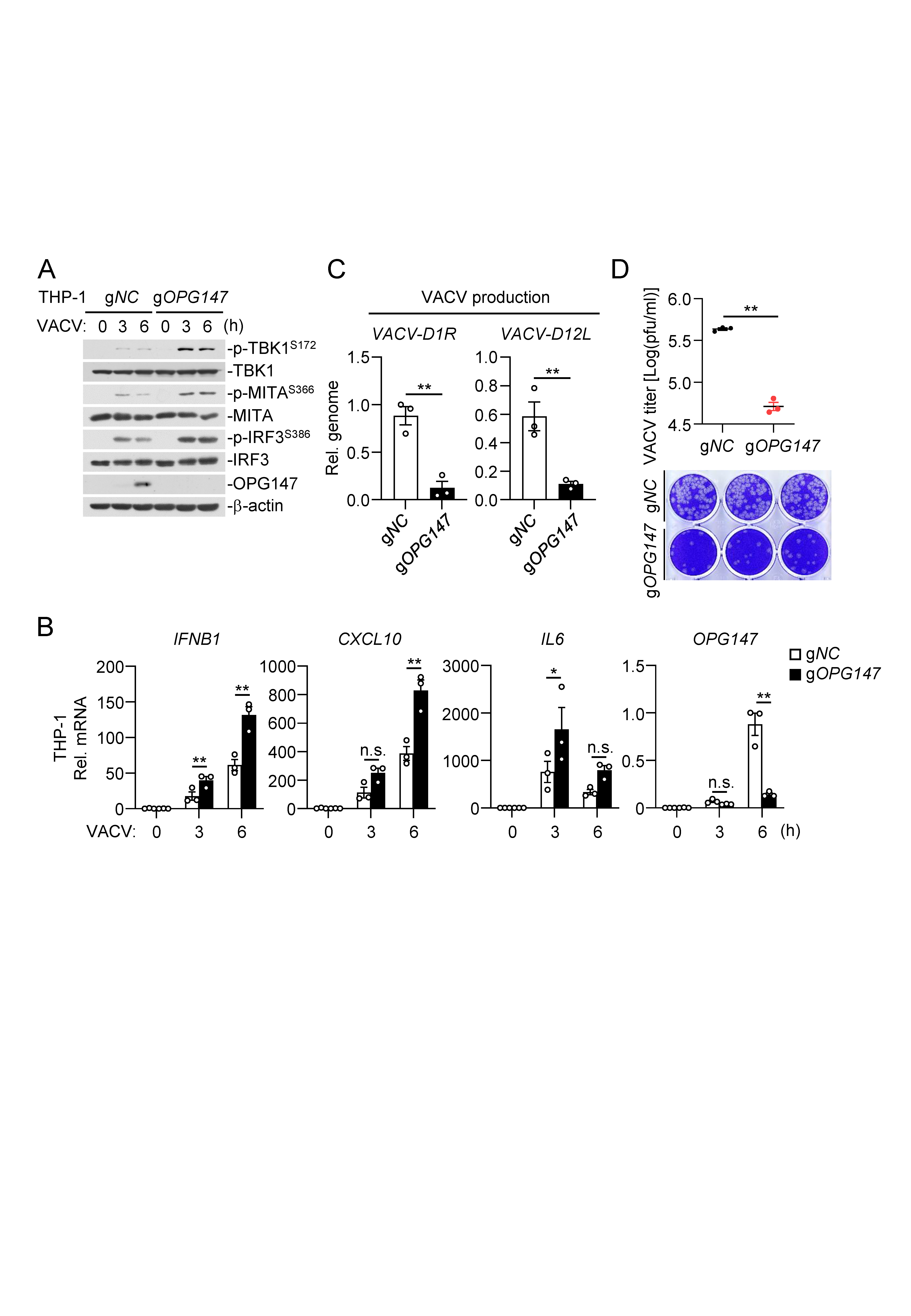

Supplement: S3 Fig — (A) Effects of OPG147-deficiency on phosphorylation of TBK1, MITA and IRF3 induced by VACV. The control and OPG147 gRNA-edited THP-1 cells (1 × 10^6^) were left uninfected or infected with VACV (MOI = 1) for the indicated times before immunoblotting analysis with the indicated antibodies. (B) Effects of OPG147-deficiency on transcription of downstream genes induced by VACV. The control and OPG147 gRNA-edited THP-1 cells (1 × 10^6^) were left uninfected or infected with VACV (MOI = 1) for the indicated times before qPCR analysis. (C&D) Effects of OPG147-deficiency on production of progeny viruses. The control and OPG147 gRNA-edited THP-1 cells (1 × 10^6^) were infected with VACV (MOI = 0.1) for 2 hours. The cells were then washed twice with PBS, cultured in RPMI 1640 medium containing 2% FBS for 48 hours. The cells were then centrifuged, and the cell culture medium was collected. Titers of progeny viruses in the cell culture medium were quantified by qPCR (C) and plaque assays (D). Data shown in (B), (C) & (D) are represented as mean ± SEM, n = 3 independent samples. All the experiments were repeated for at least two times with similar results. n.s., not significant. * P < 0.05, ** P < 0.01. (TIFF) [file ppat.1013198.s003.tiff]

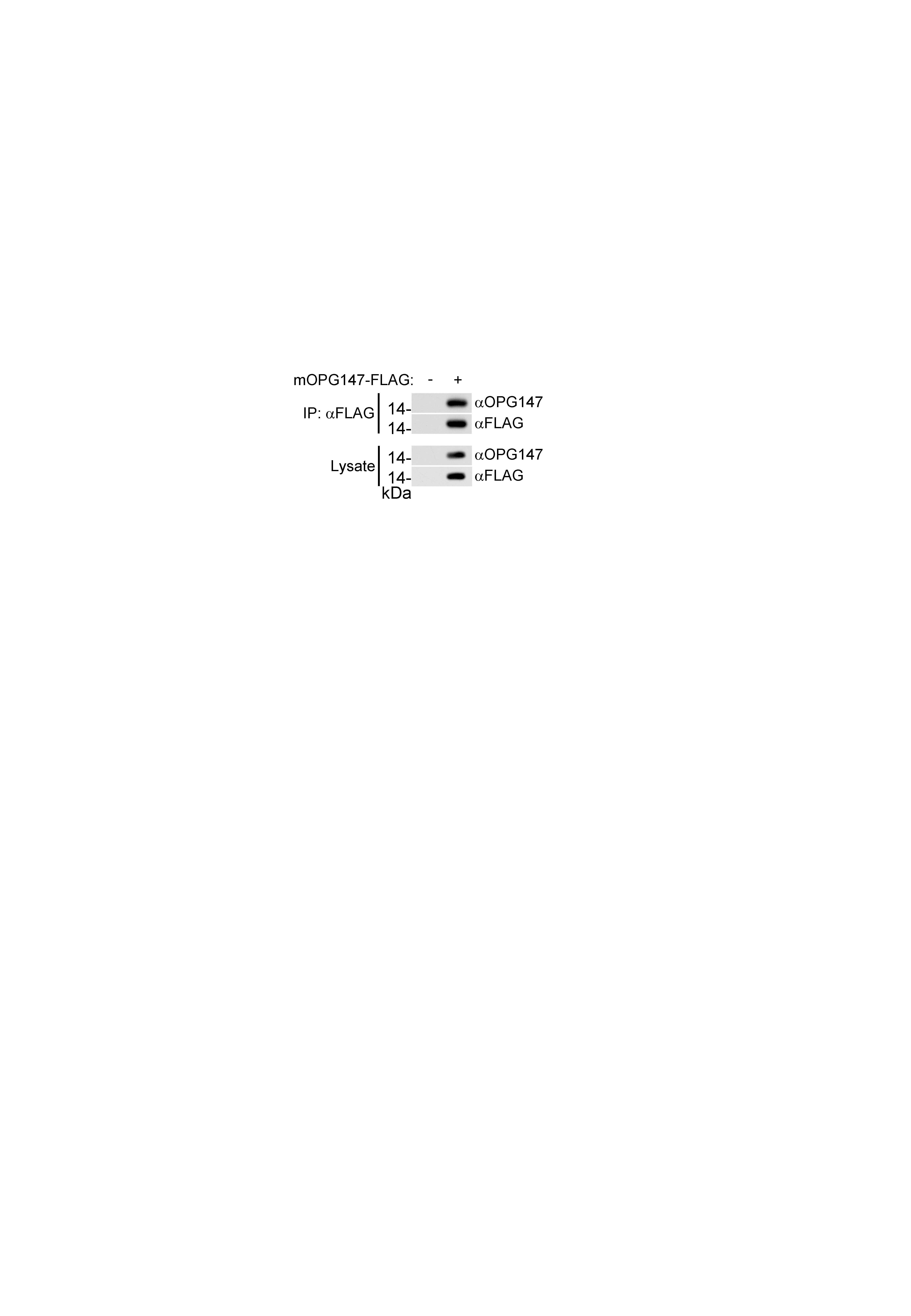

Supplement: S4 Fig — HEK293 cells (5 × 10^6^) were transfected with expression plasmids for mOPG147-FLAG or empty vector. Eighteen hours post-transfection, co-immunoprecipitation was performed with anti-FLAG. The immunoprecipitates and lysates were analyzed by immunoblotting with the indicated antibodies. (TIFF) [file ppat.1013198.s004.tiff]

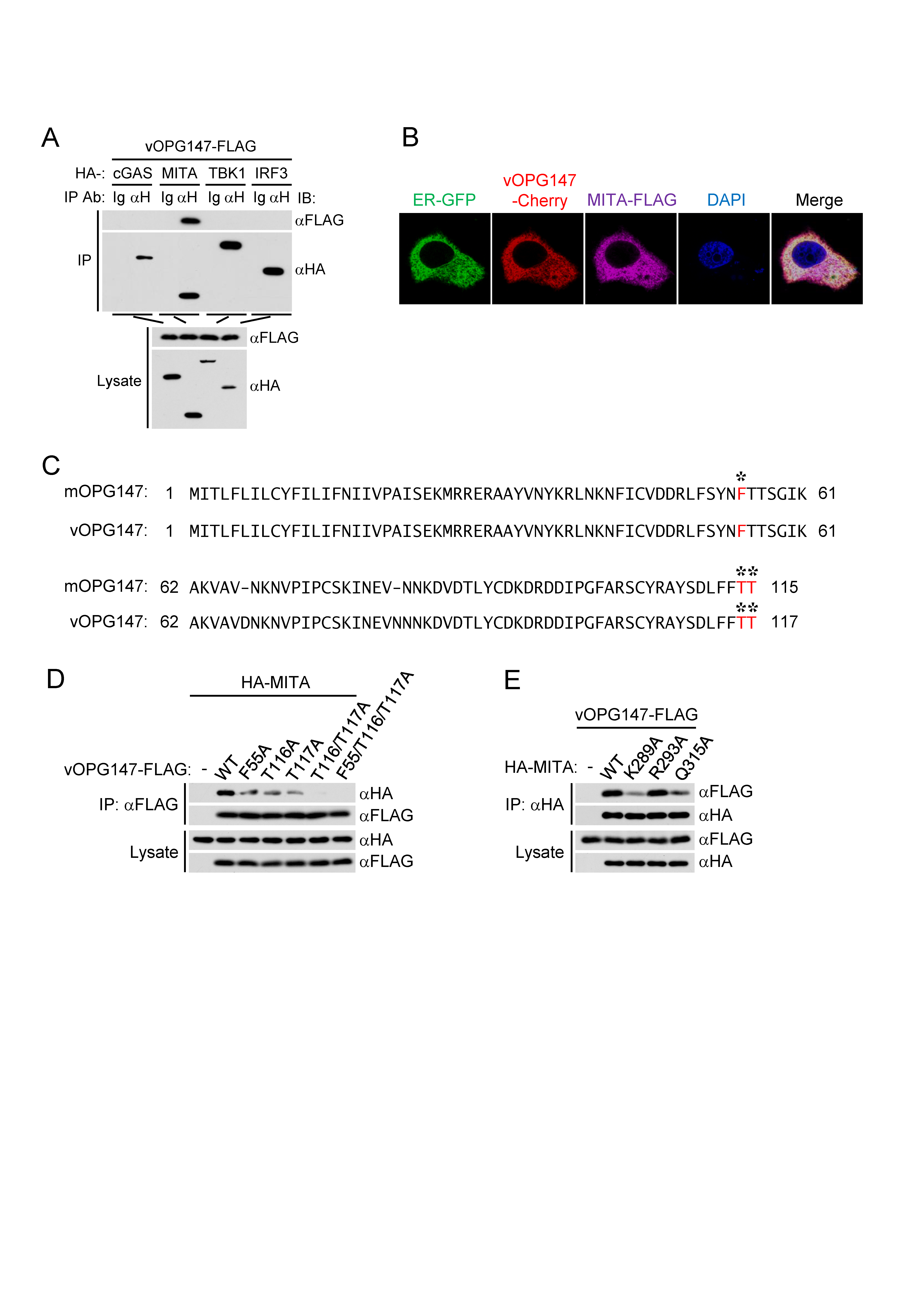

Supplement: S5 Fig — (A) vOPG147 is associated with MITA. HEK293 cells (5 × 10^6^) were transfected with the indicated plasmids. Eighteen hours post-transfection, co-immunoprecipitation was performed with control mouse IgG or anti-HA. The immunoprecipitates and lysates were analyzed by immunoblotting with the indicated antibodies. (B) vOPG147 is colocalized with MITA in the ER. Confocal microscopy of HT1080 cells transfected with ER-GFP, vOPG147-Cherry and MITA-FLAG for 24 hours. (C) Alignment of OPG147 from MPXV and VACV. Key residues involved in its interaction with MITA are highlighted in red and marked with asterisks. (TIFF) [file ppat.1013198.s005.tiff]

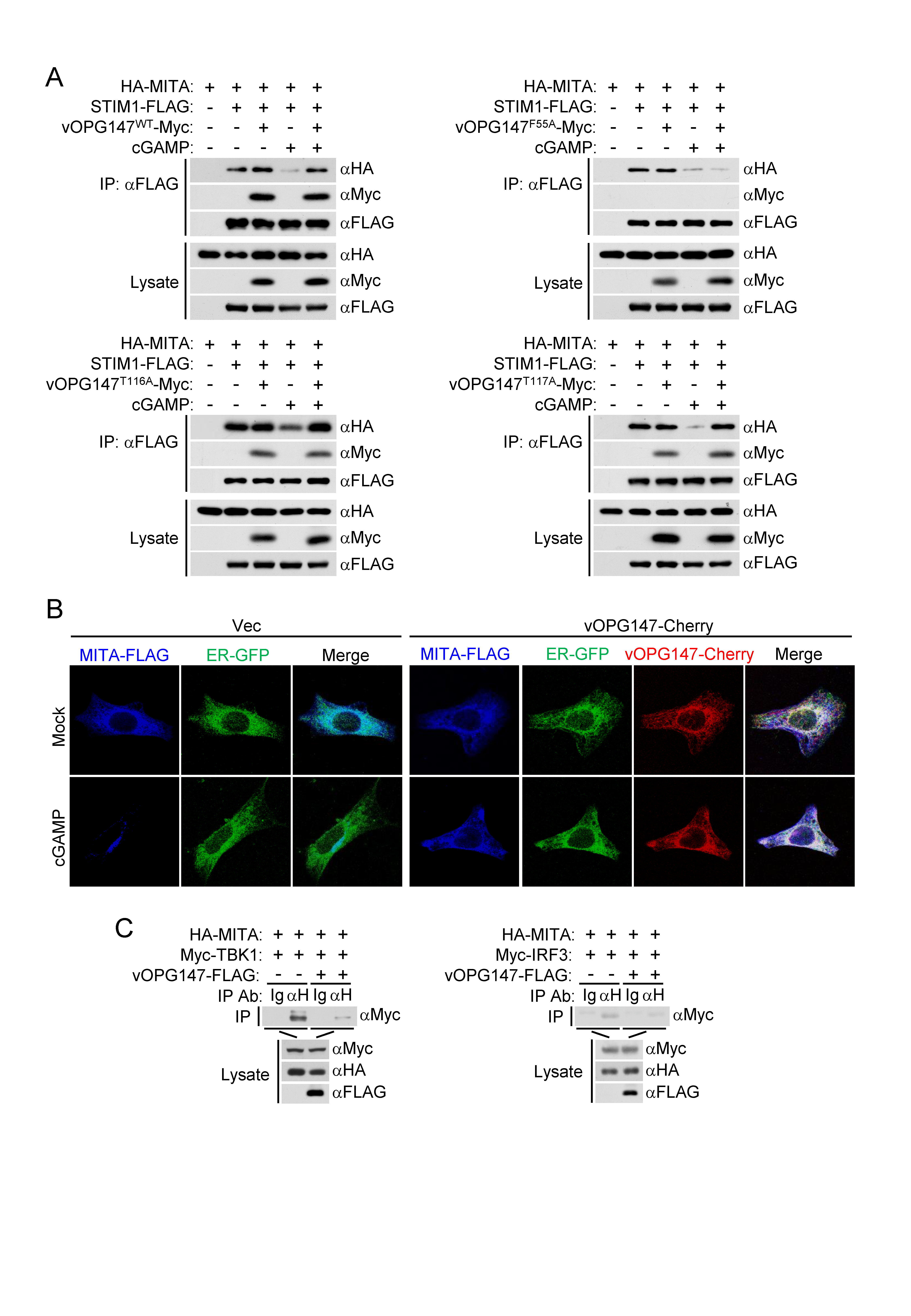

Supplement: S6 Fig — (A) Effects of vOPG147 and its mutants on association of MITA with STIM1. HEK293 cells (2 × 10^6^) were transfected with the indicated plasmids for 18 hours. The cells were untreated or treated with 2′3′-cGAMP (1 mM) for 30 min and further incubated in medium for 1 hour before co-immunoprecipitation and immunoblotting analysis were performed with the indicated antibodies. (B) vOPG147 impairs MITA trafficking. Mita-/- MLFs reconstituted with MITA-FLAG, ER-GFP and vOPG147-Cherry were stimulated with 2′3′-cGAMP (100 ng/mL) for 2 hours before confocal microscopy. (C) vOPG147 impairs recruitment of TBK1 and IRF3 to MITA. HEK293 cells (5 × 10^6^) were transfected with the indicated plasmids for 18 hours before co-immunoprecipitation and immunoblots were performed with the indicated antibodies. (D) Association between MITA and vOPG147 or its mutants. HEK293 cells (2 × 10^6^) were transfected with the indicated plasmids for 18 hours before co-immunoprecipitation and immunoblotting analysis with the indicated antibodies. (E) Association between vOPG147 and MITA or its mutants. HEK293 cells (2 × 10^6^) were transfected with the indicated plasmids for 18 hours before co-immunoprecipitation and immunoblotting analysis with the indicated antibodies. All the experiments were repeated for at least two times with similar results. (TIFF) [file ppat.1013198.s006.tiff]

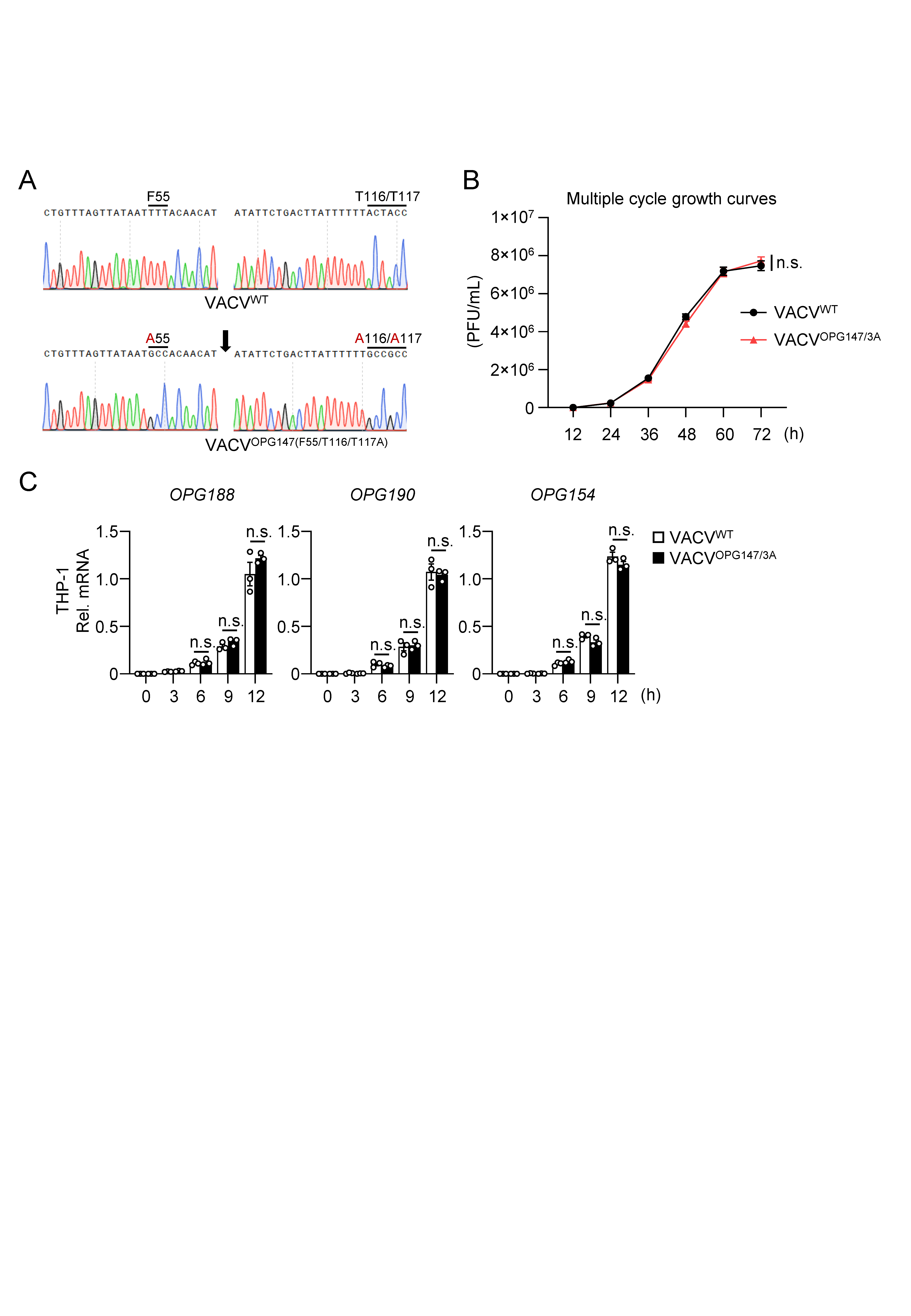

Supplement: S7 Fig — (A) Sequencing traces confirmed alanine substitutions at residues F55, T116, and T117 of OPG147 in VACV. The recombinant VACV was verified by PCR amplification of its genomic DNA followed by DNA sequencing. (B) Multiple cycle growth curves of wild-type VACV and VACVOPG 147/ 3A in Vero cells. Vero cells (1 × 10^6^) were infected with wild-type VACV or VACVOPG 147/ 3A (MOI = 0.05) for 2 hours. The cells were washed twice with PBS and cultured in MEM medium containing 2% FBS. The cells and cell culture medium were harvested at the indicated time points, subjected to three freeze-thaw cycles, and then quantified for viral titers by plaque assays using BS-C-1 cells. (C) Effects of mutation of vOPG147 F55, T116 and T117 on transcription of entry and early genes of VACV. THP-1 cells (1 × 10^6^) were left uninfected or infected with wild-type VACV or VACVOPG 147/ 3A (MOI = 1) for the indicated times before qPCR analysis. Data are represented as mean± SEM, n = 3 independent samples. n.s., not significant. (TIFF) [file ppat.1013198.s007.tiff]
